# Risk of psychological distress in parents of preterm children in the first year: evidence from the UK Millennium Cohort Study

**DOI:** 10.1136/bmjopen-2015-007942

**Published:** 2015-12-18

**Authors:** Claire Carson, Maggie Redshaw, Ron Gray, Maria A Quigley

**Affiliations:** Policy Research Unit in Maternal Health and Care, National Perinatal Epidemiology Unit, University of Oxford, Oxford, UK

**Keywords:** preterm birth, MENTAL HEALTH, depression, mother, father

## Abstract

**Objective:**

To assess whether the parents of babies born preterm (PT; <37 weeks completed gestation) are at excess risk of psychological distress (PD) at 9 months postpartum, and to explore the influence of the degree of prematurity.

**Design and participants:**

Data were drawn from the UK Millennium Cohort Study, a nationally representative prospective cohort of babies born in 2000–2002. 12 100 families with complete data available for both parents at recruitment (9 months postpartum) are included.

**Exposure and outcome:**

Mothers report of gestational age at birth (in weeks) was grouped into: very PT (<32 weeks), moderately PT (32–33 weeks), late PT (34–36 weeks), early term (37–38 weeks), full-term (39–41 weeks), post-term (42 weeks). PD was assessed using a modified Rutter Malaise Inventory, a validated instrument that has been used in both men and women to assess levels of anxiety and distress.

**Results:**

Overall, 7% of families reported a PT birth; 12.1% of mothers and 8.9% of fathers showed signs of PD at 9 months postpartum. The mothers of very PT infants had an increased risk of PD, compared with the mothers of full-term babies (unadjusted OR 2.10 (1.30 to 3.39; adjusted OR 1.66 (1.02 to 2.69)). Mothers of moderate or late PT babies had no apparent increased risk of PD. However, mothers of early term babies also showed a small excess risk of PD (adjusted OR 1.16 (0.99 to 1.36)). Unadjusted analysis suggested a doubling in the risk of PD in fathers of very and moderately PT babies, compared with fathers of full-term babies, which remains statistically significant after adjustment in the moderately PT group (adjusted OR1.98 (1.20 to 3.29)).

**Conclusions:**

The parents of very PT children are at an increased risk of PD at 9 months postpartum, and mothers of children born at early term also see an elevated risk compared with mothers of full-term babies.

**Strengths and limitations of this study**

- This is a nationally representative sample of over 12 000 families, with data on both the mother and father and gestational age. Gestational age was used in the analysis, rather than preterm birth, to examine whether degree of prematurity was important.
- A validated measure of psychological distress was used as the outcome, and a wide range of important confounders were considered in the analysis.
- The sample was restricted to families with both parents resident in the home at 9 months. No data were available on timing of past depressive episodes in either parent or on antenatal depressive symptoms in mothers.

## Introduction

Becoming new parents, or having another baby, while marked by excitement, is often also marked by disturbance in mood. Most commonly depressive symptoms have been measured with screening indicating that an estimated 10–18% of new mothers, depending on time of assessment, experience such symptoms.^1^ ^2^ Estimates of depression in new fathers range from 2% to 10%,^3–6^ although research in fathers is considerably more limited than in mothers. New parents also commonly experience heightened anxiety, which can manifest with or without depressive symptoms.^7^ ^8^ Estimates vary, but suggest that up to 11% of new mothers show symptoms of anxiety.^9^ There is some suggestion that anxiety during early parenthood is overlooked in the diagnosis of postnatal depression (PND), and that a broader diagnosis of ‘postnatal mood disorder’ may be appropriate. The term ‘psychological distress’ (PD) encompasses symptoms of depression and anxiety, with measures often also including somatic symptoms of these conditions,^10^ ^11^ and this paper focuses on this broader construct of parental PD.

It is recognised that stressful life events can trigger PD,^10^ ^12^ yet the epidemiological evidence regarding parents’ mental health following preterm birth (PTB) is sparse. PTB, defined as birth before 37 completed weeks of gestation, is undeniably a stressful experience for parents.^13^ It may involve exposure to situations in which it is feared that the baby might die, together with high levels of uncertainty and separation. PTB can occur for a variety of reasons, which may be broadly classified as either medically indicated (induction of labour or planned caesarean section) or spontaneous (spontaneous onset, or premature rupture of membranes). Medically indicated PTB is usually associated with either ill health of the mother or fetal distress and, as such, there may be increased anxiety for both parents. Not only is PTB itself stressful and anxiety-provoking, but parenting a preterm baby can also bring its own challenges. Survivors of PTB have an increased risk of short-term neonatal morbidities and of neurosensory, cognitive and behavioural problems in childhood and adulthood than their term-born counterparts, and as a result the impact on the infant and on their parents can be long-lasting.^14^ ^15^

In both mothers and fathers, the literature provides conflicting evidence with some reporting a negative effect of PTB on mental health,^16–18^ and others finding no effect.^19^ ^20^ However, previous studies have often had methodological issues including relatively small sample size,^21^ ^22^ lack of a comparison group,^22^ or presented combined data on mothers and fathers,^19^ and the evidence for fathers is particularly limited.^16^ ^23–25^ The focus has also been on extremely preterm (<28 weeks) or extremely low birthweight infants (<1000 g), and the effects across the spectrum of prematurity has not been adequately explored.

PD, and in particular depression, is known to have a negative impact on the individual, their relationships and family life,^3^ ^26^ and may have long-term implications for the development of their child, adversely affecting both cognitive and psychosocial development, and behaviour.^27–29^ In depressed parents, this negative impact is thought to act via a reduction in responsive parenting behaviours and reduced quality of the parent–child relationship.^30^ ^31^ Where only one parent is depressed (more commonly the mother), the influence of the other parent can act as a buffer and over-ride most of the detrimental effect. Where both parents are depressed, the child is at far greater disadvantage and poorer outcomes have been observed in children up to the age of 7.^32^ Heightened parental anxiety may result in adverse outcomes for the child, who is also put at increased risk of anxiety.^33^ Given that children born preterm are already at some disadvantage in comparison to their peers born at term, an increase in the prevalence of PD among this group of parents could compound the negative impact of an early delivery on child outcomes.

For these reasons, it is necessary to examine whether the mothers and fathers of preterm children are at greater risk of PD beyond the neonatal period, and whether the effect differs by degree of prematurity. This study explores how the extent of prematurity impacts on subsequent PD in both mothers and fathers in the first year, through secondary analysis of existing data from the UK Millennium Cohort Study (MCS).

## Methods

### Study population

The Millennium Cohort Study is a nationally representative prospective cohort study of 18 552 families across the UK. A random two-stage sample of all infants born in 2000–2002 and resident in the UK at 9 months was drawn from Department of Social Security (DSS) Child Benefit Registers. Ethnically diverse and disadvantaged areas were oversampled to ensure adequate representation. Children were identified by the DSS responsible for administering the benefit, who then sent a letter and information leaflet to the selected families giving them an opportunity to opt-out of the survey. Ethical approval for the survey, including the use of opt-out procedures for consent, was granted by the South West Medical Research Ethics Committee (MREC/01/6/19). Interviews with both parents were conducted when the cohort children were 9 months old, on average, and captured sociodemographic and health information, including details of depression and current mood. The cohort study does not cover births where the infant died within the first 9–10 months after birth, but these constituted less than 1% of all births. In total, 18 495 (99.6%) biological mothers and 13 167 (71%) of partners provided details about their children and about themselves, through computer-assisted questionnaires and interviews.

### Psychological distress

The main outcome of interest is symptoms of PD. This analysis includes those families where both parents completed the modified Rutter Malaise Inventory (RMI) as part of the self-administered questionnaire 9 months after the birth of their baby, and who have complete data for covariates (12 100 families, 92% of those with a partner response). The RMI was developed as a self-completion scale in simple language to cover one underlying latent construct of ‘emotional disturbance’,^34^ which ‘differentiates moderately well between individuals with and without psychiatric disorder’. The instrument has been used in studies of the general public and high-risk groups, such as carers (usually mothers).^11^ ^35^ Subsequent work has suggested that the scale comprises two dimensions—psychological malaise and physical malaise.^36^ A short 9-item instrument was developed for use in large surveys, selecting items from the original 24-item Malaise Inventory through principal components analysis,^37^ the selected items are also the highest scoring questions reported in a previous factor analysis.^36^ The nine-item modified RMI scale has been shown to correlate well with both reported depression and anxiety. The state that it identifies is described as ‘PD’, comprising aspects of both depression and anxiety.

This modified RMI scale includes yes/no questions about nine affective and physical symptoms of depression and anxiety. An overall score was calculated, and a dichotomous outcome was derived, where modified RMI score >4 indicates signs of depression or PD (threshold based on MCS literature^38^). In addition, we examined the prevalence of a self-reported medical diagnosis of depression and self-report of current treatment for depression. Biological mothers were also asked if since the birth of their child ‘there [has] ever been a time lasting two weeks or more when [they] felt low or sad?’

### Explanatory variables

The main risk factor in this analysis is PTB, defined as gestational age at birth less than 37 completed weeks. Gestational age was reported by the natural mother, and has previously been validated against linked Hospital Episodes Statistics data.^39^ Implausible values were excluded, by assessing reported birth weight and gestational age against standard growth percentiles (N=90 excluded). To examine whether the risk of PD increased with greater prematurity, we grouped births as very preterm (<32 weeks), moderately preterm (32–33 weeks), late preterm (34–36 weeks), early term (37–38 weeks), full-term (39–41 weeks) and post-term (≥42 weeks). In the analyses, full-term babies (39–41 weeks) were used as the comparison group.

Logistic regression was used to obtain ORs separately for mothers and fathers, first unadjusted and then adjusted for confounding factors. The following variables were considered as potential confounding factors:

*Socioeconomic and demographic variables*: age of parents, relationship status (cohabiting, married), educational attainment (based on highest equivalent National Vocational Qualification (NVQ)), highest socioeconomic class of the partnership, currently employed, ethnicity;

*Pregnancy and perinatal variables*: unplanned pregnancy (planned, unplanned and happy, unplanned and unhappy), mode of delivery (vaginal, instrumental, planned section, emergency section), parity (first birth or not), singleton or multiple pregnancy, any breast feeding for 4 months or longer (reflecting the recommendation at the time of survey). Birth weight was not included, as it is highly correlated with the main outcome, prematurity.

Items were considered as potential confounding factors if they were independently associated with the outcome, and remained statistically significant at the 5% level when added to the multivariable logistic regression model.

All analyses were conducted in Stata V.13,^40^ using survey weights to account for sampling and non-response. All means, proportions and ORs that are presented are weighted.

## Results

### Characteristics of the study population

In total, 12 100 families are included in the analysis, of whom: 1% of babies were very preterm (<32 weeks, n=119), 1% were moderately preterm (32–33 weeks, n=121), 5% late preterm (34–36 weeks, n=646), 20% early term (37–38 weeks, n=2381) and 69% were full-term (39–41 weeks, n=8404). Three per cent of babies were reported as delivered post-term (42 weeks, n=426). The characteristics of the study population are described in table 1.

**Table 1:** Characteristics of the parents, (all figures are weighted % unless stated otherwise)

	All	Very preterm	Moderately preterm	Late preterm	Early term	Term	Post-term
Gestational age (weeks)	23–42	<32	32–33	34–36	37–38	39–41	42
N (weight %)	12 100 (100)	119 (100)	121 (100)	646 (100)	2381 (100)	8404 (100)	426 (100)
**Sociodemographic factors—household level**							
Marital status—married	71.9	64.9	71.0	67.8	73.6	71.8	72.2
Socioeconomic position							
Mgt & prof	51.5	41.2	47.9	47.5	50.5	52.3	51.4
Intermediate	18.4	17.4	20.1	17.2	18.9	18.3	19.1
Routine and manual	22.8	26.4	17.7	25.8	23.3	22.4	22.7
Never worked/unemployed	7.3	15.0	14.3	9.5	7.3	6.9	6.8
**Sociodemographic factors—mothers**							
Age, years (mean)	29.7	29.0	30.0	29.7	30.0	29.6	29.8
Educational status							
Higher (NVQ 4+5)	38.5	34.8	31.4	35.6	36.1	39.6	38.6
Medium (NVQ 3)	14.8	11.0	12.4	11.7	14.7	15.2	15.1
Lower (NVQ 1+2)	36.6	35.4	48.7	41.9	38.0	35.6	35.7
Overseas/ other/none	10.1	18.8	7.4	10.8	11.2	9.6	10.6
Ethnicity—BME	8.0	16.3	9.4	7.1	9.0	7.7	6.8
Currently employed*	57.7	62.9	54.5	56.4	56.0	58.4	55.1
**Sociodemographic factors—fathers**							
Age, years (mean)	32.3	31.7	32.8	32.4	32.5	32.3	31.7
Educational status							
Higher (NVQ 4+5)	37.8	28.6	40.4	35.2	37.6	38.1	38.8
Medium (NVQ 3)	15.8	13.0	14.7	16.2	15.1	16.2	12.9
Lower (NVQ 1+2)	34.2	33.6	31.6	34.6	35.0	33.7	37.4
Overseas/other/none	12.2	24.8	13.3	14.0	12.3	11.9	10.9
Ethnicity—BME	8.5	13.3	12.1	7.4	8.9	8.3	9.2
Currently employed*	91.1	82.4	85.7	87.4	91.4	91.4	91.1
**Pregnancy/perinatal factors**							
Unplanned pregnancy	33.9	45.5	44.6	37.5	33.9	33.3	33.4
Mode of delivery							
Vaginal	66.0	34.2	35.5	50.5	56.0	71.1	64.7
Instrumental	11.0	4.6	7.4	11.0	7.1	12.1	14.3
Planned section	10.1	5.2	10.3	11.1	26.4	5.6	4.6
Emergency section	12.9	56.0	46.7	27.5	10.5	11.2	16.4
Singleton	98.5	88.4	82.6	89.1	97.4	99.8	99.9
Breast fed ≥4 months	35.9	18.8	24.7	27.0	31.8	37.9	42.0
First time mother	40.6	39.6	42.8	43.5	40.0	40.6	39.4
First time father	40.1	40.0	42.9	55.9	51.2	45.0	51.2
Father present at birth	94.1	79.4	81.4	90.7	93.2	94.9	95.6

*Currently employed or self-employed, a small proportion of the mothers are on leave (n=129). BME, Black and Minority Ethnic.

The average age of parents was 29.7 years for mothers and 32.3 years for fathers, and there was little variation over the gestational age groups. About half of all families (51.5%) were in the highest socioeconomic group which is higher than for the MCS overall as our study population was restricted to families in which both parents lived together and responded to the modified Rutter questionnaire. However, within our study population, a greater proportion of the families of very preterm babies was of lower socioeconomic status and had parents without educational qualifications. Overall, 8.5% of fathers and 8% of mothers described themselves as Black and Minority Ethnic group (BME), but the proportions were higher in the very and moderately preterm groups (16.3% and 9.4% in mothers, and 13.3% and 12.1% in fathers, respectively). The prevalence of unplanned pregnancies rises with increasing prematurity. Emergency caesarean section was reported in 12.9% of births, but this was markedly different across the groups, with 56.0% of the very preterm being delivered in this manner, compared with 11.2% in the full-term. Elective caesarean section was seen in 10.1% of all births, but was significantly more common in the early term group, where 26.4% of mothers reported an elective section delivery.

Table 2 summarises the characteristics of the cohort babies for the families in this analysis. Multiple births account for just 1.5% of the births in the study population overall, but 11.6% of the very preterm and 17.4% of the moderately preterm. Unsurprisingly, increasing prematurity was also associated with admission to the neonatal special care or intensive care unit, longer postnatal hospital stay (for the baby) and more frequent hospital readmission after postnatal discharge. Breastfeeding rates also varied across the groups, with the highest prevalence seen in the term and post-term groups (37.9% and 42.0%, respectively, breast feeding for 4 months or more).

**Table 2:** Characteristics of the cohort children, weighted per cent unless otherwise stated

	All	Very preterm	Moderately preterm	Late preterm	Early term	Term	Post-term
Gestational age (weeks)	23–42	<32	32–33	34–36	37–38	39–41	42
Male*	50.9	51.4	49.3	62.9	53.5	51.9	51.4
Cohort birth is twin or triplet	1.5	11.6	17.4	10.9	2.6	0.2	0.1
Timing of interview (mean, months after birth)†	9.7	9.8	9.7	9.7	9.7	9.7	9.7
Birth weight, mean, kg	3.4	1.2	2.0	2.6	3.2	3.5	3.7
Labour complications (any)	34.8	62.0	58.3	38.1	30.5	34.9	37.7
Health problems at birth or in first week (any)	25.7	89.1	81.6	59.2	28.7	20.7	23.0
Baby sent to special care‡	19.4	98.2	97.3	60.1	19.5	10.3	12.0
Age at discharge from hospital, in weeks (median, p25–p75)§	0.1 (0.3, 0.6)	8.6 (5.0, 12.9)	3.0 (2.0, 4.0)	1.0 (0.4, 1.7)	0.4 (0.1, 0.7)	0.3 (0.1, 0.6)	0.3 (0.1, 0.6)
Subsequent hospital admissions (any)	13.3	46.8	23.1	19.2	15.2	11.9	10.1
Subsequent hospital admissions (3 or more)	0.8	8.4	4.7	2.5	0.9	0.5	0.7

*Child's chronological age, not corrected age.

†Sex of first child where cohort families have twins or triplets.

‡Proportion of babies for whom data are available (because they reported health problems at birth or in first week).

§Age in weeks the baby came home from hospital, longest stay reported for multiples.

### PD and the association with PTB

#### In mothers

In total, 12.1% of mothers showed signs of PD at 9 months postpartum; the prevalence of PD was highest in the very preterm group, but dropped in the moderately preterm group and remained lower over the rest of the spectrum, as shown in table 3.

**Table 3:** Psychological distress in mothers and fathers at 9 months after birth

	All	Very preterm	Moderately preterm	Late preterm	Early term	Term	Post-term
Gestational age (weeks)	23–42	<32	32–33	34–36	37–38	39–41	42
N (weight %)	12 100 (100)	119 (1)	121 (1)	646 (5)	2381 (20)	8404 (69)	426 (3)
**Mothers psychological well-being**							
Modified Rutter Malaise Inventory >4 (signs of psychological distress)	12.1	21.7	11.6	12.2	13.7	11.7	9.4
Current treatment for depression	8.0	14.6	8.9	9.4	9.0	7.6	7.3
Ever diagnosed with depression	23.2	41.2	32.9	26.0	24.6	22.2	21.0
Felt low or sad for ≥2 weeks since birth	30.5	65.1	48.3	32.8	32.0	29.3	28.1
**Fathers psychological well-being**							
Modified Rutter Malaise Inventory >4 (signs of psychological distress)	8.9	16.1	16.8	9.1	9.3	8.6	7.5
Current treatment for depression	2.3	5.3	1.1	1.7	1.8	2.4	2.7
Ever diagnosed with depression	9.1	18.1	9.4	8.7	10.2	8.7	8.2

The unadjusted analysis suggests that the mothers of infants born at less than 32 weeks (very preterm) were more than twice as likely to be showing signs of PD at 9 months, compared with the mothers of the babies born at term (OR 2.10 (1.30 to 3.39), p<0.05). This statistically significant effect persisted after adjustment for confounding, although it was reduced (OR 1.66 (1.02 to 2.69), p<0.05). However, there was no evidence that the mothers of moderate or late preterm babies had an increased risk of PD. In unadjusted analysis, the mothers of the early term babies were also at a small increased risk of PD (OR 1.19 (1.02 to 1.40), p<0.05), and the effect remained similar after adjusting for confounding (fully adjusted OR 1.16 (0.99 to 1.36), p=0.06), as shown in table 4.

**Table 4:** ORs for psychological distress in mothers and fathers, by gestational age at birth of their child

Gestational age groups (weeks)	N	Crude association (1)	+Socioeconomic and demographic variables (2)	+Child characteristics (3)	+Pregnancy or perinatal factors (4)
Mother's psychological distress at 9 months					
<32	119	2.10 (1.30 to 3.39)	1.83 (1.12 to 2.99)	1.74 (1.08 to 2.80)	1.66 (1.02 to 2.69)
32–33	121	0.99 (0.54 to 1.83)	0.95 (0.52 to 1.72)	0.88 (0.48 to 1.59)	0.83 (0.46 to 1.49)
34–36	649	1.05 (0.80 to 1.38)	1.00 (0.76 to 1.33)	0.95 (0.73 to1.25)	0.93 (0.71 to 1.22)
37–38	2381	1.19 (1.02 to 1.40)	1.17 (1.00 to 1.37)	1.16 (0.99 to1.36)	1.16 (0.99 to 1.36)
39–41	8404	1.00	1.00	1.00	1.00
>42	426	0.79 (0.53 to 1.18)	0.77 (0.52 to 1.15)	0.77 (0.52 to 1.15)	0.77 (0.52 to 1.15)
Father's psychological distress at 9 months					
<32	119	2.03 (1.18 to 3.49)	1.63 (0.92 to 2.86)		1.58 (0.89 to 2.81)
32–33	121	2.13 (1.27 to 3.56)	2.04 (1.24 to 3.38)		1.98 (1.20 to 3.29)
34–36	649	1.06 (0.77 to 1.44)	0.98 (0.71 to 1.36)		0.97 (0.71 to 1.35)
37–38	2381	1.09 (0.90 to 1.31)	1.08 (0.89 to 1.31)		1.08 (0.89 to 1.31)
39–41	8404	1.00	1.0		1.00
>42	426	0.86 (0.59 to 1.25)	0.87 (0.60 to 1.26)		0.87 (0.60 to 1.26)

Models for fathers: 1=paternal malaise inventory; >4, gestational age groups; 2=1+father's age, relationship status, household socioeconomic class, father currently working, fathers qualifications; 3=no change; 4=2+unplanned pregnancy.

Models for mothers: 1=maternal malaise inventory; >4, gestational age groups; 2=1+ethnicity, maternal qualifications, household socioeconomic class; 3=2+multiple birth; 4=5+unplanned pregnancy.

#### In fathers

Fewer fathers than mothers showed signs of PD, just 8.9% overall. Prevalence was highest in the very preterm (16.1%) and moderately preterm (16.8%) groups (see table 3). In the unadjusted analysis, fathers of very and moderately preterm babies were twice as likely to be suffering from PD, as the fathers of term babies. After taking confounding into account, the effects are reduced but remain statistically significant in the moderately preterm group (OR 1.60 (0.91 to 2.81), p=0.11) and OR 1.97 (1.18 to 3.27), p=0.008, respectively). No statistically significant effects were observed in the late preterm, early term or post-term groups, as shown in table 4.

PD is highly associated in mothers and fathers in this cohort, so that in families where mothers are showing signs of PD, fathers are more than twice as likely to also be in the PD category (OR 2.22 (1.87 to 2.66), p<0.05). However, in absolute terms, the overlap is small—in 80.9% of families neither parent showed PD, in 10.2% only the mother and in 7.0% only the father was in the PD category, and only 1.9% of families (n=277) had both parents in the PD category.

### Reported blues, depression and treatment

While 30.5% of all mothers reported feeling low or sad for 2 weeks or more since the birth, this figure was 65.1% in the very preterm and 48.3% in the moderately preterm. The mothers of the very preterm group were also more likely to be receiving current treatment for depression, although numbers are relatively small, hence this outcome is not further explored. Only 2.3% of fathers reported that they were currently receiving treatment for depression, but again the prevalence was higher in the most preterm group (5.3%; see table 3).

## Discussion

Effects observed in the parents of very preterm babies suggest a doubling in the risk of PD in both mothers and fathers of children born at this gestational age (<32 weeks). This increase in risk in the very preterm group is consistent with the sparse literature describing the association between gestational age and parent's mental health, where others have also suggested that degree of prematurity is an important factor for maternal depressive symptoms.^41^ Suggested antecedents of PD include a trigger event resulting in a stress (fight or flight) response, symptoms (eg, fatigue), perceived loss of control and ineffective coping.^10^ This may fit the pattern of parents who experience a very preterm baby leading to an increased risk of PD, and this PD may result in symptoms that would more commonly be recognised as symptoms of postnatal depression or mood disorder (such as anxiety, depression, withdrawal from others and hopelessness).

The adverse effect of having a very preterm baby is likely to act through a number of mechanisms, so that it is partly the shock of having a very early delivery and partly the longer term stress of having a preterm infant who is more likely to be in poor health in their first year. Studies of parents of PTB have identified high rates of post-traumatic stress disorder, resulting from the unexpectedly early transition to parenthood before parents had adequate time to prepare either mentally or practically. Very preterm babies are also likely to need complex interventions including ventilation and incubation, to spend more time in neonatal care, and to have early feeding difficulties, all of which are very stressful for parents.^42^ This physical separation from their baby when they may feel unable to even touch their child is a further source of parental anxiety.^43^ In addition, the most preterm babies are more likely to experience longer term health problems—this can be seen in this study population, where the preterm babies are more frequently hospitalised again in the first 9 months—which means that there is an ongoing impact on the parents caring for a sick child, travelling to hospital or doctors’ appointments, and potentially an adverse effect on work life too. Fathers of moderately preterm children are also at increased risk of PD, whereas mothers do not appear to be. The reasons for this are not clear, and the role of chance should be considered. Outside the hospital, life continues and fathers often have to return to work and care for older children while their preterm baby is still in care, and while their partner is still in hospital. The trigger for PTB is often unknown, but is thought to be due to either poor health of the baby or of the mother. Where the mother is ill, this is an additional source of anxiety for both parents, but perhaps particularly adds pressure on the father. It is plausible that some of the individuals in our study population who had experienced symptoms of depression since the birth of their child had already received successful treatment for symptoms of PD (such as depression or anxiety), and so were not identified by the PD tool conducted at 9 months. In the first year, the support and monitoring of mental health is focused on the mother via health visitors, and so it seems likely that fewer of the distressed fathers will have been identified and treated. This may also explain some of the discrepancy in the results for men and women—the proportion of men who report receiving treatment for depression is particularly low in the moderately preterm group (1.1%, compared with 8.9% of the mothers). It may also be that the most common symptoms in PD differ between mothers and fathers, so that it is anxiety that is greater in the men (which would therefore account for the discrepancy between PD and treatment for depression).

Our results show that mothers who delivered their babies in the early term group are also at a slight increased risk of PD, compared with those in the term group. However, here it seems less plausible that it is as a result of the stress of dealing with a very ill baby, as in general children born at this gestational age are healthy. The mechanism may instead act through the mother's own health or later pregnancy complications.^44^ The higher rate of planned caesarean section in the early term group (26%) may support this hypothesis, as in cases of pre-eclampsia, poorly controlled gestational diabetes or placenta praevia, for example, it was common in the UK in 2001–2002 to deliver the mother as soon as the pregnancy reaches term. Unfortunately, we were not able to investigate in detail timing of pregnancy complications in this cohort, and so could not check if this was the case for the early term group here.

The fact that no effect was observed in the late preterm (and in the mothers, the moderately preterm) may be partly explained by the step change in infant health that occurs at about 32 weeks’ gestation. Prior to this, the increase in the risk of severe morbidity (such as retinopathy of prematurity^45^ and cerebral palsy^46^) and other neurodevelopmental problems is much higher. By dividing up the preterm population, we were able to look at how the degree of prematurity influences parental outcomes. Other studies where there has been no observed effects of PTB on parents’ PD (including depression and/or anxiety) have often dichotomised gestation as <37 weeks or not,^20^ and in this instance it seems likely that the absence of an association at later gestational ages dilutes the observed effect, and so conclude that prematurity has little impact on parents’ later well-being. This highlights the importance of not treating ‘preterm’ as one homogeneous group, as it encompasses a real spectrum of risk as gestational age decreases.

### Strengths and limitations

This study of over 12 000 families draws on a nationally representative cohort study with a good response rate, and employed weights to account for non-response and design effects due to stratified sampling. The effects of new fatherhood are often overlooked but we analysed data on both mothers and fathers in cohabiting relationships, so that the effects on both parents could be assessed. However, as the MCS did not collect data on non-resident fathers, our study does not include lone parents for whom the impact of a PTB or sick infant may be even more detrimental due to a lack of support from a partner.

Detailed maternal report on delivery allowed us to look at the effects across the continuum of gestational age. Gestational age and birth weight were validated against hospital records, and implausible values were removed, so that we are confident in the gestational age groups reported here. Relatively small numbers of cases in the moderately and very preterm groups does mean that the effects seen here should be treated with caution, although the results are consistent with the findings of other studies.^16–18^

The MCS used the modified RMI in the survey at 9 months, which is a validated measure of PD; the instrument has been used in a number of cohort studies.^47^ ^48^ PD was assessed at 9 months by which time some of those who have displayed symptoms of PD will have been identified and treated. However, other studies have suggested that after an initial increase in the first 8 weeks postpartum, the prevalence of depressive symptoms remains remarkably constant in the first year.^49^ PD is not equivalent to depression but is strongly correlated, and includes the anxiety component that is also an important aspect of mental well-being. Prevalence of PD was approximately 9% in men in our population, which is similar to the 10% observed in fathers at 9 months postpartum in an American cohort also born in 2001.^30^ PD encompasses symptoms of both depression and anxiety, and it is possible to assess PD in a self-completed survey. However, we cannot separate those who are depressed from those who are anxious, and greater detail may have helped to explain some of the observed patterns and the differences seen in mothers and fathers.

In women, antenatal depression is associated with PTB,^50^ and also increases the risk of postnatal depression,^51^ and so it is possible that the observed association is due to reverse causality. In the MCS survey, data on past depression was collected without detailed information on timing or number of past episodes. Therefore, positive responses could include antenatal depression, depression diagnosed since the birth but before the survey, or indeed describe the current depressive episode. As a consequence, we were unable to control for a history of depression in our analysis. However, the results indicate that both fathers and mothers are at increased risk of depression in the very preterm group, and father's depression is unlikely to trigger a PTB. It is also possible that women who are anxious at 9 months postpartum were also anxious in pregnancy, and this group may be more likely to request an elective caesarean—resulting in the observed increased risk in this group. Data on other important confounding factors were collected, and so we could assess a range of socioeconomic and demographic confounding factors. Although PTB clearly precedes the assessment of PD, other potentially useful data (eg, relationship quality, child temperament, parent's health behaviours such as alcohol consumption) were assessed concurrently with PD, and so we also chose to exclude these possible confounders and mediators because the temporal relationship was unclear. This is a limitation of cross-sectional survey data, and future studies of PTB and PD would greatly benefit from data collected periodically throughout pregnancy and the first year.

## Conclusions

This study suggests that both mothers and fathers of very preterm babies are approximately twice as likely to experience symptoms of PD at 9 months postpartum, and though this effect is reduced after adjusting for confounding factors, it does not disappear. Fathers in the moderately preterm group also show an increased risk of PD, but this is not mirrored by the mother's risk. A novel finding is that mothers in the early term group are also at a small increased risk of PD: this should be a focus for future work as the early term group represents a large proportion of all births, and so the impact of this small increase in risk on maternal well-being at the population level will be greater. Small numbers in the most preterm groups, and a lack of data on antenatal depression, mean that the estimates should be treated with caution but, in general, the results for the preterm groups are in line with the existing, sparse literature. Significantly, the findings suggest that *both* parents of very preterm babies are at risk of PD. Further research on the impact of PTB of the mental health of parents is needed, so that there can be increased vigilance for symptoms of PD in this group. Where symptoms are identified early and support can be given, the negative impacts of PD on the individual and their family could be reduced.
